# Pulmonary evaluation of whole-body inhalation exposure of polycarbonate (PC) filament 3D printer emissions in rats

**DOI:** 10.1080/15287394.2024.2311170

**Published:** 2024-02-06

**Authors:** Mariana T. Farcas, Walter McKinney, W. Kyle Mandler, Alycia K. Knepp, Lori Battelli, Sherri A Friend, Aleksandr B. Stefaniak, Samantha Service, Michael Kashon, Ryan F. LeBouf, Treye A. Thomas, Joanna Matheson, Yong Qian

**Affiliations:** a National Institute for Occupational Safety and Health, Morgantown, WV, USA; b Pharmaceutical and Pharmacological Sciences, School of Pharmacy, West Virginia University, Morgantown, WV, USA; c Office of Hazard Identification and Reduction, U.S. Consumer Product Safety Commission, Rockville, MD, USA

**Keywords:** Thermoplastics, thermal decomposition, printer emissions, 3D printer emitted nanoparticles, volatile organic compounds (VOC), inhalation toxicology, pulmonary toxicity, systemic markers

## Abstract

During fused filament fabrication (FFF) 3D printing with polycarbonate (PC) filament, a release of ultrafine particles (UFPs) and volatile organic compounds (VOCs) occurs. This study aimed to determine PC filament printing emission-induced toxicity in rats via whole-body inhalation exposure. Male Sprague Dawley rats were exposed to a single concentration (0.529 mg/m^3^, 40 nm mean diameter) of the 3D PC filament emissions in a time-course via whole body inhalation for 1, 4, 8, 15, and 30 days (4 hr/day, 4 days/week), and sacrificed 24 hr after the last exposure. Following exposures, rats were assessed for pulmonary and systemic responses. To determine pulmonary injury, total protein and lactate dehydrogenase (LDH) activity, surfactant proteins A and D, total as well as lavage fluid differential cells in bronchoalveolar lavage fluid (BALF) were examined, as well as histopathological analysis of lung and nasal passages was performed. To determine systemic injury, hematological differentials, and blood biomarkers of muscle, metabolic, renal, and hepatic functions were also measured. Results showed that inhalation exposure induced no marked pulmonary or systemic toxicity in rats. In conclusion, inhalation exposure of rats to a low concentration of PC filament emissions produced no significant pulmonary or systemic toxicity.

## Introduction

Additive manufacturing (AM) is a broad manufacturing term that encompasses a range of processes that create objects by adding material through a computer-aided design model (Plessis, Preez, and Stefaniak 2022; Stefaniak, Du Preez and Du Plessis, 2021b). Three-dimensional (3D) printing is a form of AM, which builds objects through layer-by-layer deposition of feedstock material using a 3D printer machine and computer software (Plessis, Preez, and Stefaniak 2022) Fused filament fabrication (FFF), also known as Filament Freeform Fabrication), is one 3D printing process in which filaments are melted and extruded from a heated nozzle to deposit material. FFF is an emerging technology and one of the most popular additive manufacturing processes, especially for consumers and small manufacturers such as industrial, academic military and residential sectors (MacCuspie et al. 2021).

Polycarbonate (PC) is a versatile material, and PC filaments are widely used for FFF 3D printing. Polycarbonate filaments are often loaded with additives to achieve different properties of the print objects. These additives range from dyes, photopolymer resins, organometallic compounds, carbon nanomaterials, nanometal oxides to micro-meter-scale particles such as copper, bronze, steel, tungsten, gold, and aluminum nitride (Vance et al. 2017). Several engineered nanomaterials were infused into PC filaments, such as silicon dioxide nanoparticles, titanium nitride nanoparticles (Vidakis et al. 2021), titanium carbide nanopowder (Vaisanen et al. 2022; Vidakis, Petousis, Grammatikos, et al. 2022), aluminum nitride nanoparticles (Vidakis, Petousis, Mangelis, et al. 2022), and carbon nanotubes (Potter et al. 2021).

During heating, PC filaments undergo thermal degradation and release fine particles (0.1 to 2.5 um) and ultrafine particles (d < 100 nm) as well as numerous volatile, and semi-volatile organic compounds (VOCs) that may be derived from PC polymer and additives in the polymer (Alijagic et al. 2022; Azimi et al. 2016; Byrley et al. 2020; Gu et al. 2019; Stefaniak et al. 2017, 2019; Tedla et al. 2022). These emissions may pose a potential hazard to human health. Currently, the potential health hazard attributed to PC filament printing emissions exposure remains to be determined.

A NIOSH research group used a condensation nuclei counter to study PC filament emission rates, and determined that the number-based particle emission rates from an industrial-scale material extrusion AM machine were approximately 2.2 × 10^11^ number/min and the total VOCs emission rates were approximately 19 mg/min (Stefaniak et al. 2019). In addition, Stefaniak et al. (2019) found low levels of acetone, benzene, toluene, and *m,p*-xylene during PC filament printing processes. Potter et al. (2019) noted that PC filament emissions contained bisphenol A (BPA), phenol, chlorobenzene, DEHP, and di-tert-butylphenol. Previously, Farcas et al. (2019) examined PC filament printer emission-induced cell toxicity originating from a commercial PC 3D printer which was generated in a chamber using a 3D printer and subsequently collected for incubation in a cell culture medium. The number-based size distribution of the particles within the chamber was between 140 and 170 nm and mean particle sizes in the cell culture medium were 201 ± 18 nm. Analysis of elemental composition of particles collected in the cell culture medium found was as follows: C, O, Ca, Na, Si, Ni, Cr, Fe, S, Al, and Cl. The organic compounds in the emission collection cell culture medium identified were BPA, p-isopropenylphenol, and phenol. At 24 hr post-exposure, PC emissions were internalized in human small airway epithelial cells (SAEC) and induced the following concentration-dependent responses: cytotoxicity, oxidative stress, apoptosis, necrosis, as well as increases in number of pro-inflammatory cytokines and chemokine production in SAEC (Farcas et al. 2019). These results demonstrated that PC filament 3D printing emissions were associated with a cellular toxicity in SAEC.

Although cell-based *in vitro* toxicity analysis is increasingly applied to (1) screen and rank chemicals for prioritizing toxicity studies, as well as to (2) determine underlying toxic mechanisms, the toxicological significance of *in vitro* study-generated data in hazard and risk assessment is limited. In comparison with animal-based *in vivo* studies, *in vitro* cell investigations (1) lack tissue-specific differentiated functions, (2) physiological context with other cells and tissues, and (3) biological concordance in metabolism of xenobiotic chemicals (Blaauboer 2008). The aim of this investigation was to examine the influence of PC filament printer emission-induced acute and sub-chronic pulmonary and systemic toxicity in rats.

## Materials and methods

### Three-dimensional printer emissions inhalation exposure system

A custom-designed inhalation exposure system was used to deliver either HEPA-/carbon-filtered air or consumer-grade FFF 3D printer emissions to a whole-body rodent exposure chamber in real time (Farcas et al. 2020). Briefly, HEPA-/carbon-filtered air was drawn through the exposure system at 30 LPM using a vacuum and mass flow controller (MCRW-50-DS; Alicat Scientific, Tucson AZ). For control animals, HEPA-/carbon-filtered air was drawn directly into the temperature-controlled (22–24°C) exposure chamber; for emissions-exposed animals, HEPA-/carbon-filtered air was drawn through an airtight chamber that housed three FFF 3D printers before arriving at the temperature-controlled exposure chamber. During exposures, the 3D printer housing chamber and the exposure chamber doors would remain closed. Emissions and conditions inside the exposure chamber were continuously monitored and controlled in real-time using external equipment that was connected to sampling ports and custom software.

### Three-dimensional printer settings for PC filament

The three FFF 3D printers (nozzle diameters = 0.4 mm) were programmed to simultaneously print a 12.7 × 12.7 × 2.54 cm object using a black PC filament over a 4-hr period. Each print job required 240 g filament and occupied most of the build plate surface. The printer nozzle temperatures were set to the recommended print temperature for PC, 300°C. The build plate surfaces were prepared the previous day using a thin coating of ABS juice made by dissolving 40 g ABS filament in 100 ml acetone, and fumes from evaporated acetone were flushed out of the chamber prior to an exposure. Printing PC parts is difficult because of its tendency to warp as it cools. This warping can detach the part from the print bed mid print. In addition to warping, PC does not adhere well to standard print beds without using some type of adhesive. Through experimentation and online searches, it was found that the most effective method to keep PC on a print bed is to use “ABS Juice.” A thin layer of the ABS juice was applied to the print bed with a paint brush and then allowed to dry. This results in a thin layer of ABS on top of the print bed which is barely visible. The ABS sticks well to the print surface, and the PC melts into the ABS and thus adheres to this layer. The PC filament was stored at room temperature in an airtight dry box when not in use.

### Three-dimensional PC emissions collection and characterization

Emissions were collected and characterized as described previously (Farcas et al. 2020). A brief overview is as follows: A Data RAM (DR-1500; Thermo Electron Co., Waltham, MA) was used to continuously monitor aerosol mass concentrations inside the exposure chamber; readings were verified daily by gravimetric analysis (37 mm diameter, 0.45 μm pore-size Teflon filters, 2 LPM). Each exposure maintained a mean aerosol mass concentration of 0.529 mg/m^3^ for 4 hr. Real-time particle counts were also recorded using a condensation particle counter (CPC; Model 3787, TSI Inc., Shoreview, MN) and associated software.

A fast mobility particle sizer (FMPS; Model 3091, TSI Inc., Shoreview, MN) was used to collect particle size data in 5-sec intervals during several 4-hr mock exposures without animals in the exposure chamber. Aerosols were also collected onto track-etched polycarbonate filters (25 mm, 0.1 μm pore-size, 1 LPM for 20 min) for particle morphology analysis by field emission-scanning electron microscopy (FE-SEM; Hitachi S-4800, Tokyo, Japan). In accordance with NIOSH Manual of Analytical Method (NMAM) 3900 (NIOSH 2018), emissions were sampled using fused silica-lined, evacuated canisters (450 mL; Entech Instruments Inc., Simi Valley, CA) with 3-hr capillary flow controllers, and then analyzed for specific VOCs in accordance with NIOSH Method 3900 using an Entech 7200/7650 pre-concentration system coupled with an Agilent 7890/5975 gas chromatograph-mass spectrometer (GC-MS; Santa Clara, CA). Samples were collected (1/day, 5 different days) during 4-hr mock exposures without animals in the exposure chamber; mean concentration and standard deviation were calculated for individual VOCs.

### Animals

Male Sprague-Dawley [Hla: (SD) CVF] (SD) rats (6–7 weeks old, weighing 200–225 g) were purchased from Hilltop Lab Animals (Scottdale, PA), housed 4 per cage in ventilated polycarbonate cages, and acclimated for a minimum of 7 days prior to starting the study. The facility provided a controlled environment with HEPA-filtered air, 22 ± 2°C temperatures, and 40–60% humidity. The animals were fed irradiated Teklad 2918 (Harlan, Madison WI) and provided with an ALPHA-dri^®^/Teklad sani-chips bedding mix and tap water *ad libitum*. The CDC-Morgantown Institutional Animal Care and Use Committee (accredited by AAALAC International) reviewed and approved the study protocol.

### Experimental design

Each of the 60 acclimated animals was assigned to one of the two treatment groups, air control or 3D printer emissions exposed, for an exposure duration of 1, 4, 8, 15, or 30 days (*n* = 6 per exposure group). For 4 hr/day, 4 consecutive days/week, air control groups were subject to whole-body inhalation of HEPA-/carbon-filtered air, while 3D printer emissions-exposed groups were subject to whole-body inhalation of real-time emissions from FFF 3D printers printing with PC filament. Rats were sacrificed 24 hr post-exposure via intraperitoneal (ip) pentobarbital injection (100–200 mg/kg) (Fort Dodge Animal Health; Fort Dodge, IA) followed by exsanguination via whole-blood collection from the abdominal aorta. Whole blood was immediately transferred to two collection tubes: one EDTA-containing vacutainer for whole blood hematological analysis and one clot activator-/polymer gel-containing vacutainer for serum chemistry analysis (Becton-Dickinson; Franklin Lakes, NJ). Bronchoalveolar lavage (BAL) fluid (BALF) samples were collected while the left lung was clamped, and the right cardiac lobe was tied off. The non-lavaged right cardiac lobe and lavaged right lobes were collected and stored at −80°C; the left lung and head/nasal tissues were preserved for histopathological evaluation.

### Three-dimensional PC filament emission particle deposition estimates in nasal passages, tracheobronchial and alveolar regions

The Multiple-Path Particle Dosimetry (MPPD) model (Anjilvel and Asgharian 1995) was used to estimate PC-emissions particle deposition mass for the head/nose, tracheobronchial, and alveolar regions, and collective deposition for tracheobronchial and alveolar regions under conditions with and without clearance. Particle deposition mass without clearance: FMPS data was converted to mass distribution using the assumption of spherical particle shape with a density of 1.3 g/cm^3^ (density of PC). Animal breathing rate (120 breaths/min), tidal volume (1.7 ml), 4-hr exposure time, and 0.5 mg/m^3^ were the parameters used for the MPPD model. Particle deposition mass with clearance: a detailed description of these methods is provided in Farcas et al., (2020).

### Bronchoalveolar lavage fluid (BALF) analysis

A brief summary of these previously described methods by Farcas et al. (2020) is as follows:

### BALF collection and cytology

The rat’s right lung was perfused with 6 ml cold PBS via a tracheal cannula. The lung was massaged for 30 sec, cold PBS withdrawn, and the process was repeated a second time, yielding the first fraction of BALF. A second BALF fraction was collected by repeating the aforementioned process using 5 ml aliquots of PBS until a 15 ml volume was recovered. The two BALF fractions were centrifuged (800 × g, 10 min, 4°C), and the supernatant from fraction one and cell pellets from both fractions were kept for further analysis (supernatant from fraction two was discarded). The supernatant was used to measure total protein levels, lactate dehydrogenase (LDH) activity, and surfactant and cytokine levels. The pellets were combined, resuspended in 1 ml PBS (Lonza, Pearland, TX), and used for a total cell count (Beckman Coulter Multisizer 4 particle counter, Coulter Electronics, Hialeah, FL) and cell differential (HEMA-stained cytospin slides, 300+ cell-count per slide).

### Transmission electron microscopy (TEM) staining of BALF cells

BALF cells were fixed in Karnovsky’s fixative, post-fixed in 2% osmium tetroxide, mordanted in 1% tannic acid, and stained *en bloc* in 0.5% uranyl acetate. Samples were then dehydrated with ethanol, infiltrated in propylene oxide, embedded in EPON^™^, and sectioned at 70 nm. Grids were stained with 4% uranyl acetate and Reynold’s lead citrate and imaged using a JOEL 1400 transmission electron microscope (Tokyo, Japan).

### Scanning electron microscopy (SEM) images of lungs

A 5 μm non-lavaged lung section was mounted on a carbon planchet and deparaffinized with xylene. The sample was sputter-coated with gold/palladium and imaged using a Hitachi S4800 field emission scanning electron microscope (Tokyo, Japan).

### Total protein and LDH activity

A Pierce^™^ BCA Protein Assay Kit (Fisher Scientific) and an LDH Reagent Set (Pointe Scientific, Lincoln Park, MI) were used in conjunction with a Synergy H1 Microplate Reader (BioTek, Winooski, VT) to collect total protein and LDH activity data, respectively, from the BALF samples.

### Surfactant proteins a (SP-A) and D (SP-D)

SP-A and SP-D ELISA kits (Biomatik USA, Wilmington, DE) were employed in conjunction with a Synergy H1 Microplate Reader to collect surfactant protein data from the BALF samples.

### Cytokine levels

A V-PLEX Pro-inflammatory Panel 2 Rat (Immunoassay) Kit (MSD, Rockville, MD) was utilized in conjunction with a QuickPlex SQ 120 plate reader (MSD, Rockville, MD) to collect pro- and anti-inflammatory cytokine data from the BALF samples.

### Blood processing and analysis

Blood collected in EDTA-containing and clot-activator-/polymer gel-containing vacutainers was processed and analyzed as previously described in Farcas et al (2020). A brief overview is as follows:

### Characterization of blood cells and hematological parameters

A complete blood count was performed for each sample 30–45 min after collection using a ProCyte Dx Hematology Analyzer (IDEXX Laboratories, Inc., Westbrook, ME).

### Serum chemistry profile

Serum was extracted by allowing whole blood to clot at room temperature followed by centrifugation at 2,500 rpm for 10 min. Serum biochemical parameters were evaluated using a Catalyst One Chemistry Analyzer (IDEXX Laboratories, Inc., Westbrook, ME).

### Serum cytokine levels

Pro- and anti-inflammatory cytokine levels were determined according to manufacturer’s protocols using a V-PLEX Pro-inflammatory Panel 2 Rat Kit (MSD) to dilute the samples and a QuickPlex SQ 120 plate reader (MSD) to acquire data.

### Lung and nasal passages histopathological evaluation

The non-lavaged left lung was inflated with 10% neutral buffered formalin (NBF), embedded in paraffin, cut at 5 μm and stained with hematoxylin and eosin (H&E) for histopathological evaluation. Lesions were reviewed by a veterinary pathologist and classified in the following manner: WNL = within normal limits; 1 = minimal change (barely exceeds WNL); 2 = mild/slight change (lesion is identifiable but is of limited severity); 3 = moderate change (lesion is prominent with a potential for increased severity); 4 = severe change (lesion occupies the majority of the organ and is as severe as possible). After processing the lungs, the nasopharynx was flushed with 10% NBF, and nasal passages collected. Nasal tissues were fixed in formalin for 1 week, then decalcified in 13% formic acid. Standard nasal sections (T1, T2, T3, and T4) were taken (Young 1981) and embedded in paraffin, cut at 5 μm, and stained with H&E.

### Statistical analysis

All statistical analyses were performed using SAS/STAT v9.4 for Windows. Two-way analyses of variance (ANOVA) were utilized to assess treatment effects on each dependent variable. Post-hoc pairwise comparisons were performed using Fisher’s LSD. Some variables were log transformed to meet the assumptions of the analysis. If the assumptions were still not met following the transformation, then the Kruskal-Wallis’ test, followed by the Wilcoxon rank-sum nonparametric test, was utilized to assess pairwise comparisons. Differences were considered significant using a p-value <0.05.

## Results

### Three-dimensional PC filament printing emissions characterization

In this study, a custom-designed 3D printer emission generation and inhalation exposure system was applied to expose male Sprague-Dawley rats to 3D PC filament printer emissions according to the methods previously published (Farcas et al. 2020). Consumer-grade FFF 3D printers emit nano- and ultra-fine-sized particles in addition to VOCs during printing (Farcas et al. 2019). The details of the particle mass and count concentrations, particle sizes, and VOC measurements in this 3D printing emission generation and inhalation exposure system were recently published by Krajnak et al. (2023). In brief, the particle count concentration rose rapidly in under 10 min to the maximum detectable level of particles by the CPC instrument (10^6^ particles/cm^3^), then after approximately 30 min fell to a steady state value of approximately 600,000 particles/cm^3^. The mass concentration increased slower during the first 30 min of exposure, then remained between 0.4 and 0.8 mg/m^3^. The actual average mass concentration determined with gravimetric filters over all exposure days was 0.592 mg/m^3^ with a daily mean standard deviation of 0.26 mg/m^3^. A typical size distribution plot (particle count based) of the particles inside the exposure chamber during a test run was recently published by our team (Krajnak et al. 2023). Particle size data were collected every 5 sec using a fast mobility particle sizer. The mean particle electric mobility diameter was 40 nm (Krajnak et al. 2023).

GC/MS was applied to measure the VOCs in the PC filament emissions from samples collected over a 4 hr collection period. Data demonstrated that, among VOCs quantified during printing, the levels of all measured VOCs were below Occupational Safety and Health Administrations (OSHA) permissible exposure limits (PELs) (Krajnak et al. 2023). Acceptable exposure levels for receptors for consumers in the home environment and non-healthy workers may be lower. However, among these measured VOCs, the levels of acetaldehyde, acetone, ethanol, and ethylbenzene were the highest (Krajnak et al. 2023). The levels of BPA and bisphenol A diglycidyl ether were also measured in the PC filament emissions. The mean level of BPA in the emission was 5.3 ± 0.18 μg/m^3^, and no bisphenol A diglycidyl ether was detected (Krajnak et al. 2023).

**FIGURE 1.**
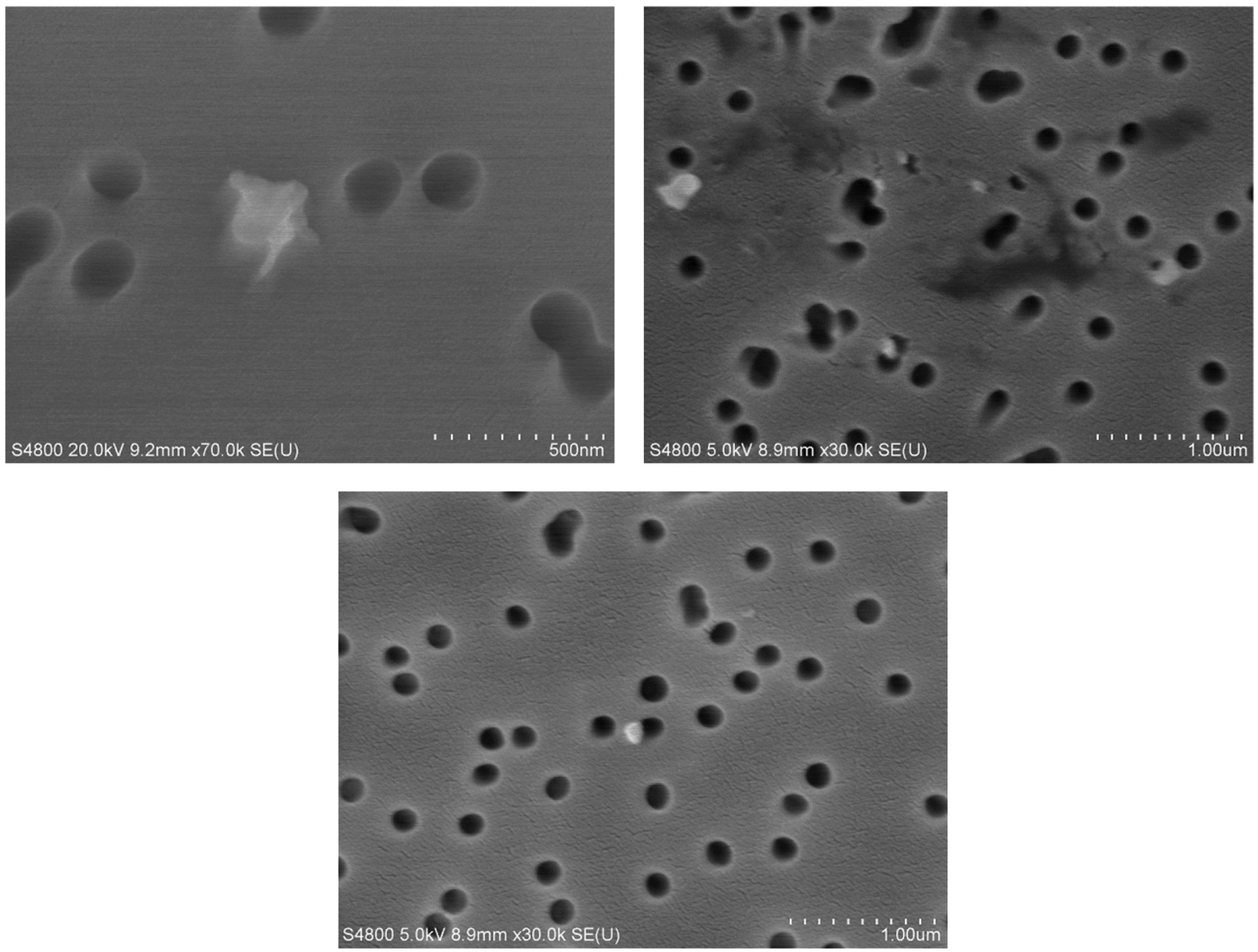
Representative images of PC 3D printer-emitted particles onto filters. The surface morphology and elemental composition were analyzed using FE-SEM.

The particles generated during FFF 3D printing were also sampled onto filters and imaged with a field-emission scanning electron microscope. Several of the particles are depicted in Figure 1. The circular dark holes are the pores in the sample filter. Typical particle physical diameters ranged from 40 nm up to 500 nm.

### Estimation of 3D PC filament emission particle deposition in the nasal passages, tracheobronchial and alveolar regions

The estimation of 3D PC filament particle deposition in the nasal passages, as well as in tracheobronchial, and alveolar regions was conducted using the MPPD model (Anjilvel and Asgharian 1995). Data for particle deposition estimates without clearance are presented in Table 1 and with clearance in Table 2.

### Pulmonary particle deposition

**FIGURE 2.**
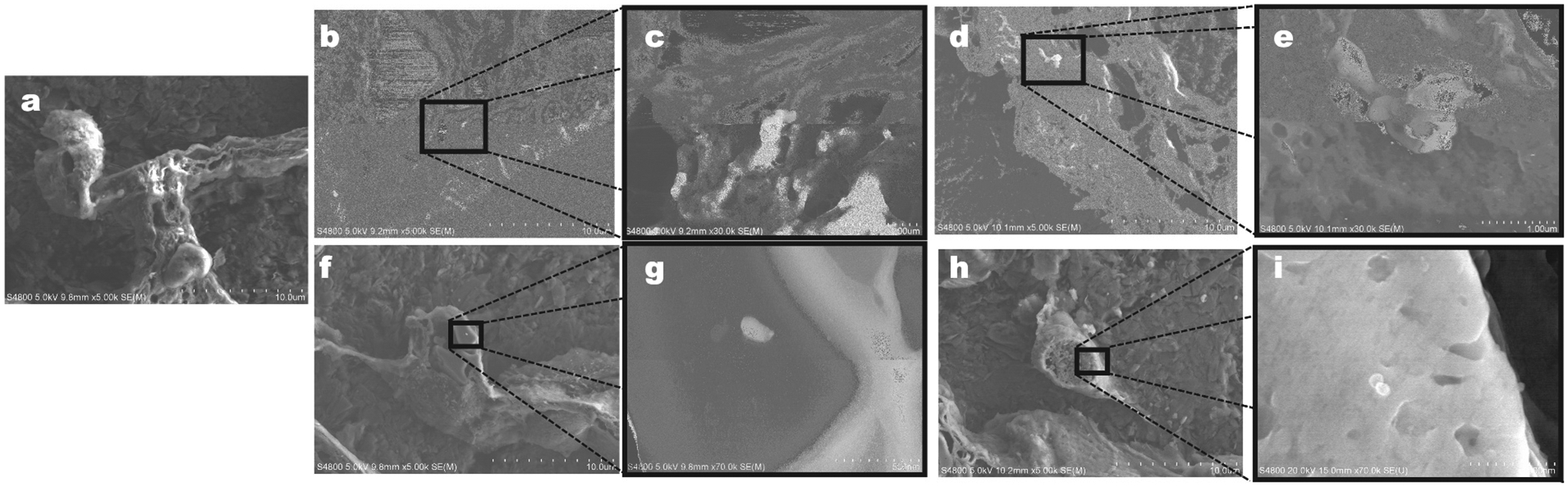
Representative images of PC 3D printer-emitted particles deposited in the alveolar region at days 1 and 30 of exposure. The images were taken using FE-SEM. A: air-control; B,C,D, and E: day 1 of exposure; F, G, H, and I: day 30 of exposure.

**FIGURE 3.**
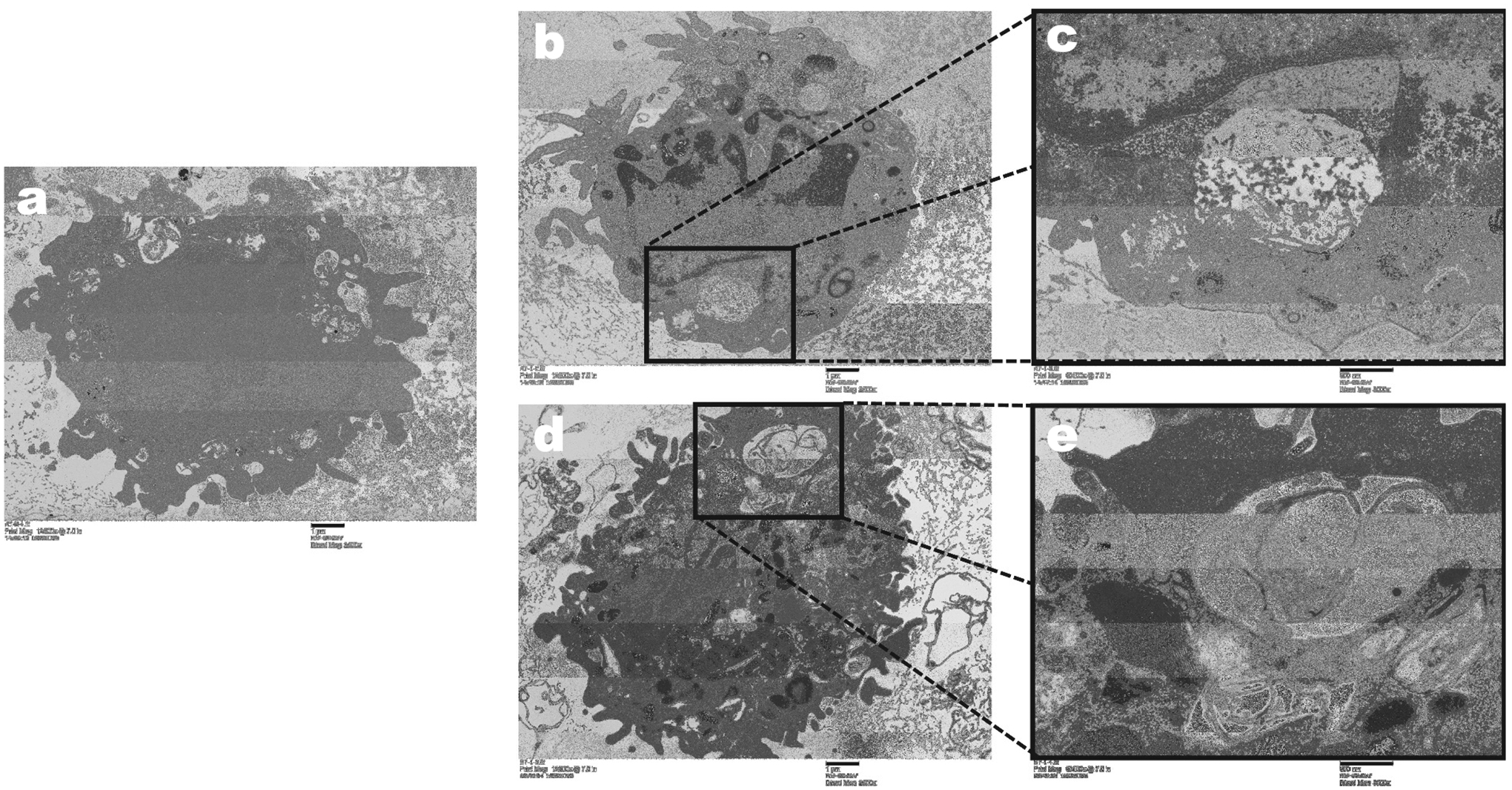
Representative images of cellular uptake of PC 3D printer-emitted particles in BAL cells at days 1 and 30 of exposure by TEM. A: air-control; B and C: day 1 of exposure; D and E: day 30 of exposure.

SEM imaging of rat lungs displayed particle deposition in the alveolar region of the one- and 30-day exposure groups as early as 24 hr post exposure and no particles were found in the control rat lungs (Figure 2), indicating that particles were able to deposit in the alveolar region of exposed rats. TEM imaging analysis was performed to determine whether the deposited particles were engulfed by BALF cells and their uptake changed the cell morphology. The TEM analysis demonstrated that the particles were able to enter alveolar macrophages in BALF cells at all durations of exposure at one-day post exposure (Figure 3, only one and 30 days of exposure are shown). Uptake of particles in the alveolar cells occurred in membrane-lined vacuoles and located in the cytoplasm. The morphological analysis demonstrated that uptake of particles did not induce any marked changes in alveolar macrophage morphology.

### Three-dimensional PC filament emission-induced pulmonary injury and inflammation

**FIGURE 4.**
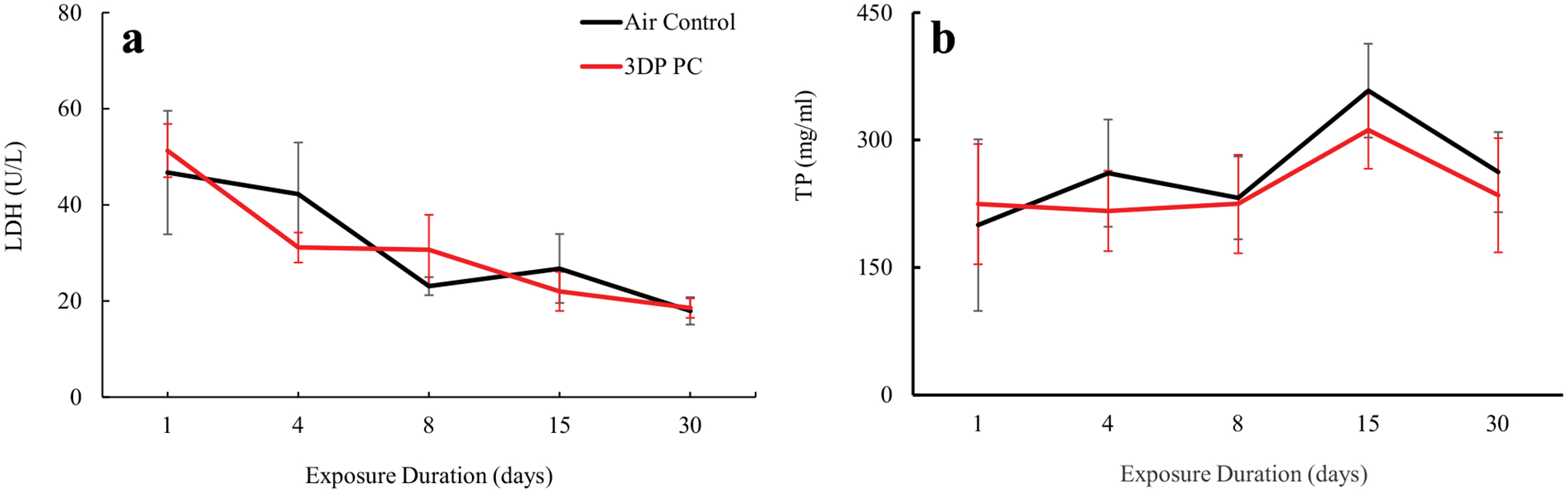
Biomarkers of pulmonary injury in BALF. A: LDH activity; B: total protein. The rats were exposed for 1, 4, 8, 15, and 30 days to air or PC 3D printer emissions and euthanized at 24 hr post last exposure. Values represents means ± SEMs; *N* = 6.

**FIGURE 5.**
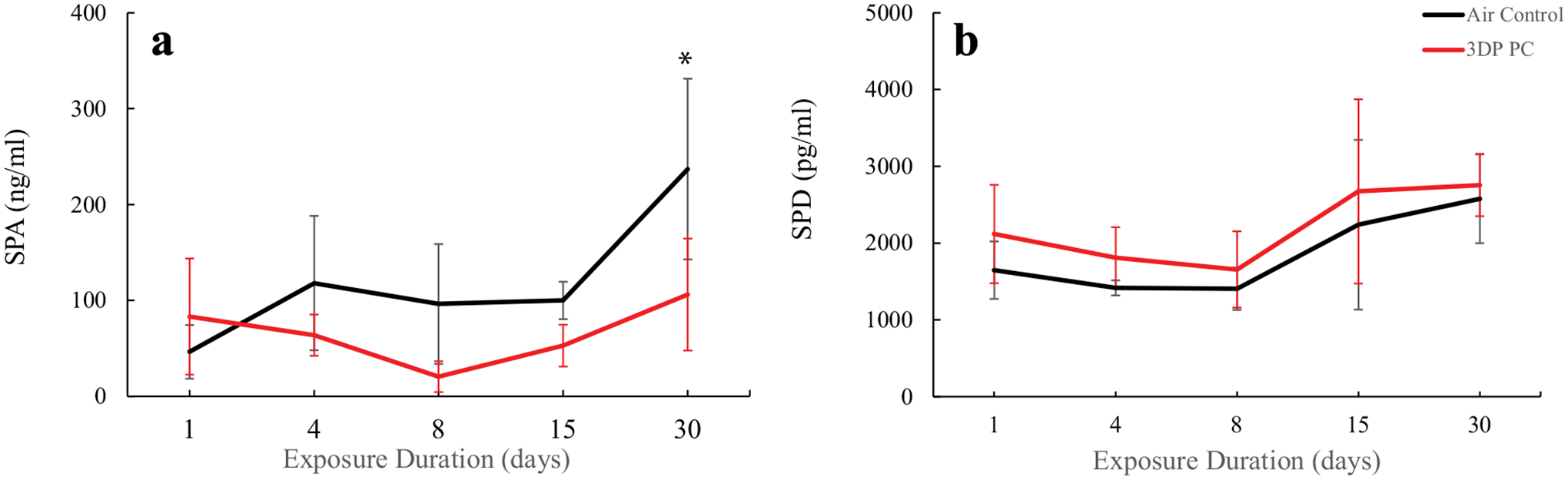
Biomarkers of alveolar epithelium injury. A: SPA; B: SPD. The rats were exposed for 1, 4, 8, 15, and 30 days to air or PC 3D printer emissions and euthanized at 24 hr post last exposure. Values represents means ± SEMs; *N* = 6.

Three-dimensional PC filament particle-induced pulmonary injury was evaluated via changes in LDH activity and levels of total protein (TP), surfactant proteins (SP)-A, and SP-D in BALF. Figure 4 illustrates that PC filament emissions produced no significant alterations in LDH activity or TP levels for all emission exposure groups compared to air-only control at all duration exposure groups. In agreement with the results of LDH and TP, no significant alterations in SP-A and SP-D were found between PC filament emission and air-control groups (Figure 5). Data indicate that 3D PC filament emissions did not significantly affect pulmonary damage in rats at the exposure level of the current study.

**FIGURE 6.**
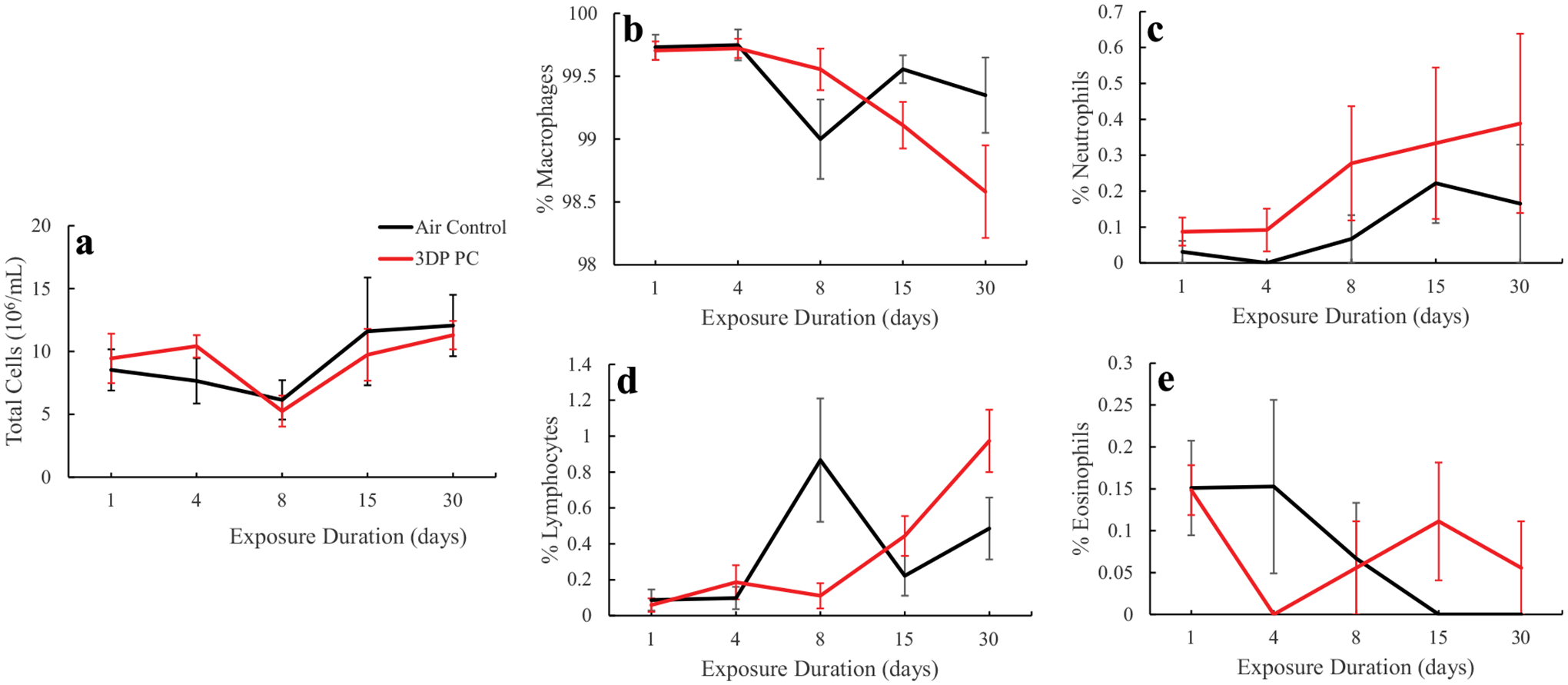
Biomarker of pulmonary damage in BAL. A: total cells; B: macrophages; C: neutrophils; D: lymphocytes; E: eosinophils. The rats were exposed for 1, 4, 8, 15, and 30 days to air or PC 3D printer emissions and euthanized at 24 hr post last exposure. Values represents means ± SEMs; *N* = 6.

To further determine 3D PC emission-induced pulmonary damage and inflammation, total cells as well as differentiated cells in the BALF were measured. Results indicated that there were no significant changes in the numbers of total cells, macrophages, neutrophils, lymphocytes, and eosinophils in BALF between air-only groups and PC filament emission-exposed groups (Figure 6), indicating that PC emissions did not produce significant pulmonary damage and inflammation in rats at these exposure doses and durations. Several key inflammation-related cytokines and chemokines in BALF were measured to assess PC filament emission-induced pulmonary inflammation. No significant alterations in IFN-γ, IL-10, IL-13, IL-1β, IL-4, IL-5, IL-6, KC, and TNF-α were observed in the PC emission exposed groups (Table 3). These results further demonstrated that PC filament emission produced no significant pulmonary inflammation in rats at the currently generated exposure level.

### Histopathology of lung and nasal passages

The routine histopathological analysis detected no marked changes in any lung or nasal passage sections among all treatment groups.

### Systemic effects

To determine 3D PC filament emission inhalation exposure-induced systemic effects in rats, blood samples were collected to measure several hematological parameters as well as biomarkers of muscle, metabolic, renal, and hepatic function. Inhalation exposure to PC filament emissions did not significantly affect hematological biomarkers, except for a few time points (Table 4). Serum biomarker and chemistry analysis demonstrated that 3D PC filament emission exposure overall exerted no marked effects on metabolic, hepatic, and kidney function in rats (Table 5). Taken together, the data demonstrated that inhalation exposure of rats to 3D PC emissions did not induce significant systemic changes at the current exposure doses.

## Discussion

In this study, the particle mass exposure concentration in the chamber calculated from FMPS measurements, assuming bulk density of PC and spherical particle shape, was between 0.3 and 3 mg/m^3^, and daily mean mass concentration from gravimetric measurements of particles collected on filters, over all exposure days, was 0.529 mg/m^3^ (Krajnak et al. 2023). Note that the effective density of the aerosol emissions is unknown but was reasonably assumed to be equivalent to bulk PC for the purpose of calculating particle mass. Determination of the effective density of airborne particles is complex; although Katz et al. (2020) determined that during FFF 3D printing with ABS, values were approximately 30% higher compared with bulk, but for PLA, the effective and bulk densities were similar. In addition, it is important to note that the composition of the particles released during 3D printing with PC filament was not analyzed in this investigation and might be made up of plasticizers, other semi-volatile constituents in the filament, and/or polycarbonate polymer. In the aforementioned study by Katz et al. (2020) used an aerosol mass spectrometer and electron energy loss spectrometry to interrogate the composition of aerosols generated from FFF 3D printing. For ABS, both techniques identified aromatic compounds and the energy loss spectra were consistent with polymeric styrene, which supports the concept that bulk composition was at least partially reflective of aerosol composition. It is conceivable that variations of particle emissions over the 4-hr print job may be associated with several factors: 1) build plates are heated and this may lead to extruded plastic cooling faster the farther away it is from the first layer; 2) the 3D printing housing chamber is not temperature controlled, and thus the air temperature inside this chamber might fluctuate during print jobs; and 3) variations of filament composition from the manufacturing process. The precise chemical composition of each roll of the filament was proprietary and not made available. Another factor that might account for the observed variability is that generating the exact same mass concentration in the exposure chamber each day was found to be difficult, partly because three printers were utilized and it was not uncommon for one of them to fail while printing. Prints might fail if (1) the part did not stay attached to the build plate, (2) the print nozzle became clogged, or (3) a software error occurred. During an exposure, if a print failed, that printer was stopped to prevent damage to the printer. If a printer door was opened to try and fix it, this might dilute the concentration down to near zero during an animal exposure, thus the door was never opened to attempt to fix a stopped printer.

The estimated particle alveolar dose over 4 days of exposures (8.1 μg) in the exposed rats can be scaled to a human equivalent dose by using known alveolar surface area values. The alveolar surface area of the human lung is approximately 70 m^2^ (Hasleton 1972; Butler 1976; Gehr, Bachofen, and Weibel 1978), and the surface area of a rat lung is approximately 0.4 m^2^ (Pinkerton et al. 1982; Stone et al. 1992). By using these values, the equivalent human alveolar lung burden was calculated; 8.1 μg × 70 m2/0.4 m2 = 1,418 μg. The human alveolar deposition fraction for the particles generated in this study was 0.2256 based on the MPPD model (Anjilvel and Asgharian 1995; Yeh and Schum 1980). By using this deposition fraction, the total inhaled particle mass was calculated: 1,418 μg/0.2256 = 6.29 mg. A healthy adult human has a minute ventilation of 8 L/min (Pritchard 1976). The total volume of air inhaled by a worker over the duration of 4 days of animal exposures is estimated: 4 days × 4 hr/day × 60 min/hr × 8 L/min = 7,680 L (7.68 m^3^). Dividing the total inhaled particle mass by the total volume of inhaled air may result in a human equivalent average exposure concentration: 6.29 mg/7.68 m^3^ = 0.819 mg/m^3^.

The particle mass concentration of 0.529 mg/m^3^ is on the same order of magnitude as reported for injection molding and grinding tasks (approximately 0.1 to 0.3 mg/m^3^) that involved heating PC polymer (Boonruksa et al. 2017). On a count basis, the mean PC particle concentration for all animal exposures during 3D printing was 4.6 × 10^6^ particles/cm^3^ (Krajnak et al. 2023) although this value was likely an underestimate because the concentration in the inhalation chamber often reached the upper limit of the FMPS instrument (1 × 10^6^ particles/cm^3^) during the first 45 min of a print. This exposure profile was consistent with test chamber investigations that reported a peak concentration of 1 × 10^6^ particles/cm^3^ at the start of 3D printing with PC filament and remained above 10^4^ to 10^5^ particles/cm^3^ for the remaining print time (Ding, Wan, and Ng 2020). Stefaniak et al. (2021a) noted that during large format additive manufacturing with PC polymer in the workplace, an average particle number concentration of 1.2 to 2.5 × 10^4^ particles/cm^3^ was detected. The count median diameter during exposures was 35 nm (Farcas et al. 2019), consistent with particle sizes observed in 3D printing test chamber studies with PC filament (Ding, Wan, and Ng 2020).

Acetaldehyde, acetone, ethanol, and ethylbenzene were released during 3D printing with PC filament and were a component of the total exposure received by animals (Krajnak et al. 2023). These VOCs are all known thermal breakdown products of PC polymer (Cabanes and Fullana 2021; Guillemot, Oury, and Melin 2017). Acetaldehyde was present in the highest concentration, which was consistent with Guillemot et al. (2017) who reported acetaldehyde as a main breakdown product of PC polymer. Acetone levels may be explained by the method of using ABS Juice for print bed preparation. ABS juice is made by dissolving 40 g of ABS print filament into 100 ml of acetone. A thin layer of the ABS juice is applied to the print bed with a paint brush and then allowed to dry. This results in a thin layer of ABS on top of the print bed which is barely visible. The ABS sticks well to the print surface, and PC melts into the ABS and thus adheres to this layer.

Most PC polymer is made from BPA monomer. When PC polymer is heated, BPA and several other thermal breakdown products are released into the air (Erickson 2007; Huang et al. 2018). Our results showed BPA is one of the many VOCs emitted from 3D PC filaments (Stefaniak, Du Preez, and Du Plessis 2021b). The mean concentration of BPA in the emissions was 5.3 ± 0.18 μg/m^3^ (Krajnak et al. 2023). MPPD estimates of BPA deposition in the entire respiratory system were 36 ng on day 1, 536 ng on day 15, and 1,072 ng on day 30 of exposure (Krajnak et al. 2023). It was suggested that PC filament emission-induced neuroendocrine toxic effects may be associated with elevated BPA in the PC filament emissions (Krajnak et al. 2023). There is little information regarding BPA-induced pulmonary and systemic toxicity *in vivo*. One study showed that whole-body inhalation exposure of BPA up to 90 mg/m^3^ for 8 weeks (6 hr/day, 5 days/week) induced no marked effect on body weight, hematology, serum chemistry, organ weights, or histopathological lesions in rats (Chung et al. 2017). O’Brien et al (2014) found that when exposed to BPA *in utero* via maternal diet, an individual may experience BPA sensitization without symptoms of pulmonary inflammation when the subject becomes an adult. Although no pulmonary nor systemic toxicity was detected in this study, it is difficult to exclude the potential toxic effects of BPA due to the relative low concentration of BPA in the PC filament emissions.

To our knowledge, there are no published studies of respiratory effects attributed to inhalation of PC polymer particles. The absence of appreciable respiratory toxicity following exposure to PC emissions during 3D printing in the current study might be explained by multiple factors. First, results obtained from this study may reflect that the respiratory tract was not a target organ. The count median diameter of PC particle emissions during 3D printing (35 nm) was sufficiently small to reach the gas-exchange region of the lung. Once deposited, these particles may translocate across lung epithelium cells to the blood, distribute systemically, and subsequently induce adverse effects in other organs. Indeed, Krajnak et al (2023) found that 30 days of PC filament emissions exposure affected the neuroendocrine system by reductions in thyroid stimulating hormone, follicle stimulating hormone and prolactin in rats, which were associated with an elevation in markers of cell injury, decrease in active mitochondria in the olfactory bulb and gonadotropin releasing hormone cells and fibers, fall in number of tyrosine hydroxylase immunolabeled fibers in the arcuate nucleus, and decrease in spermatogonium (Krajnak et al. 2023).

Second, exposure to biologically active components of PC particles might have been attenuated by the presence of polymer matrix. This effect of the polymer matrix is in agreement with findings in prior investigation that demonstrated the toxicity of engineered nanomaterials was attenuated when embedded in a polymer matrix such as paint (Saber et al. 2012; Smulders et al. 2014).

Finally, it is possible that the particle mass concentration used for exposures was simply insufficient to induce adverse respiratory health effects. If the absence of respiratory health effects was a reflection of low mass concentration, some caution is warranted in the interpretation of these results as it might mean that exposure to PC particles is not without risk. In the current study, the mean particle mass exposure (0.529 mg/m^3^) was generated by three desktop-scale 3D printers. Secondo et al. (2020) noted that some indoor spaces may have up to 30 desktop-scale 3D printers operating simultaneously, which might translate into higher particle mass exposure than what was employed in the current study and potential toxicity may have been masked.

Previously, Farcas et al. (2019) *in vitro* studies found that 3D PC filament emissions induced a concentration-dependent toxicity in human small airway epithelial cells (SAEC). Further, the PC filament emissions produced concentration-dependent oxidative stress, apoptosis, necrosis, and generation of pro-inflammatory cytokines and chemokines in SAEC. Our *in vitro* studies also indicated that exposure to 3D PC filament emissions might pose a toxicological risk. However, in this *in vivo* study, data demonstrated that inhalation exposure to 3D PC filament induced no significant pulmonary or systemic toxicity in rats. It is possible that there are several factors that may be attributed to the divergence of the toxicological observations between our *in vivo* and *in vitro* toxicity investigations, including lung burden differences and exposure conditions.

There is a significant difference in the equivalent alveolar lung burdens of PC particles between the *in vitro* and *in vivo* studies (1697 μg vs 60.6 μg). In our previous *in vitro* experiments, the mean delivered PC particle concentration was 3.64 × 10^7^ particles/ml, with a count distribution median size of approximately 120 nm in the solution. By utilizing a lognormal fit of the particle size distribution, and using a MATLAB script, the particle mass per ml of the solution was calculated as 1.4 μg/ml by assuming spherical particles with a density of 1.2 g/cm^3^. Each plate well had a surface area of 0.33 cm^2^ and was dosed with 0.1 ml of solution such that the particle mass per surface area was calculated: (1.4 μg/ml × 0.1 ml)/(0.33 cm^2^) = 0.424 μg/cm^2^. By assuming that a typical rat lung has an alveolar surface area of 4000 cm^2^, one can calculate the equivalent alveolar lung burden of 1697 μg in rats (0.424 ug/cm^2^ × 4000 cm^2^ = 1697 μg). In our current studies, the alveolar burden of day 30 exposure without and with clearance were 60.6 μg and 25.5 μg, respectively (determined with MPPD software). Note that MPPD only considers mechanical mechanisms (e.g., mucociliary escalator) when estimating lung clearance, but the actual amount cleared may be higher if removal by chemical dissolution is an important mechanism for particles released during FFF 3D printing with PC filament. Currently, an inhalation project exposing rats to higher concentrations of 3D PC filament emissions might help to elucidate the role of lung burden.

Traditional submerged cell culture technology was applied in our previous *in vitro* studies. There are several drawbacks of the submerged cell culture that may affect toxicological outcomes: a) our SAEC-based submerged cell culture technology does not mimic the physical features of airway mucosa nor alveolar unit of the lung, b) the physicochemical characteristics of the PC filament emissions may change in the cell culture media (Upadhyay and Palmberg 2018), and c) the submerged cell culture technology is unable to recapture the inhalation conditions nor the particle deposition pattern in lungs (Upadhyay and Palmberg 2018). Indeed, our previous parallel investigations of 3D ABS filament emission-induced toxicity showed a similar pattern to this study, that ABS filament emissions produced a significant toxicity in the SAEC-based submerged cell culture model but not *in vivo* pulmonary or systemic toxicity in rats (Farcas et al. 2019, 2020). However, when an Air-Liquid Interface (ALI) model of primary normal human-derived bronchial epithelial cells was applied to perform *in vitro* toxicity assessments, 3D PC filament emissions-mediated toxicity was noted indicating that the results obtained from the ALI model corresponded with those from the *in vivo* studies (Farcas et al. 2022). It is possible that an ALI model may be a better choice to evaluate 3D PC filament emission-induced *in vitro* toxicity.

Finally, for live organs *in vivo*, there are many other cells coexisting that may communicate with the targets cells to compensate emission-initiated toxicity; therefore, a single-type cell-based toxicological observation may not adequately simulate *in vivo* conditions (Madorran et al. 2020).

## Conclusions

Consumer-grade level PC filament printers emitted UFPs and VOCs during real-time printing. Data demonstrated that whole-body inhalation exposure to 3D PC filament emissions at the concentration of 0.529 mg/m^3^ for 1, 4, 8, 15, or 30 days exposure (4 hr/day, 4 days/week) did not produce significant pulmonary or systemic toxicological responses in rats, which was inconsistent with toxicological results observed in our *in vitro* studies. Given the significant lower lung burden of the 30-day exposure in rats compared with the *in vitro* study, it is warranted to perform a toxicity assessment to expose rats to a higher concentration of 3D PC filament emissions to confirm whether the concentration of 0.529 mg/m^3^ of PC filament emissions is too low to initiate any pulmonary and systemic toxic responses in rats. Currently, there is an ongoing study to determine pulmonary and systemic toxicologic responses to 2.5 mg/m^3^ of PC filament emissions via whole-body inhalation exposure in rats, and these results might provide further insights for 3D printing exposures.

## Figures and Tables

**TABLE 1.** Modeled Total Lung Burden without Clearance

Days of exposure	Particle mass deposited (μg)			
	Nose	Tracheobronchial	Alveolar	Total Lung
1	20.2	1.5	2.0	3.5
4	80.9	6.0	8.1	14.1
8	161.9	12.0	16.2	28.2
15	303.5	22.4	30.3	52.7
30	607.0	44.8	60.6	105.4

**TABLE 2.** Modeled Total Lung Burden with Clearance

Days of exposure	Particle mass deposited (μg)			
	Nose	Tracheobronchial	Alveolar	Total Lung
1	20.2	1.5	2.0	3.5
4	20.2	1.7	7.6	9.3
8	20.2	1.7	13.3	15.0
15	20.2	1.7	19.6	21.3
30	20.2	1.7	25.5	27.2

**TABLE 3.** Level of Cytokines in Bronchoalveolar Lavage Fluid

		Exposure duration (days)				
Marker	Treatment	1	4	8	15	30
IFN-γ (pg/mL)	Air	0.25 ± 0.03	0.24 ± 0.04	0.26 ± 0.00	0.44 ± 0.04	0.40 ± 0.03
	PC	0.23 ± 0.01	0.25 ± 0.02	0.28 ± 0.02	0.43 ± 0.03	0.43 ± 0.02
IL-10 (pg/mL)	Air	0.48 ± 0.03	0.48 ± 0.04	0.46 ± 0.01	0.71 ± 0.03	0.68 ± 0.04
	PC	0.47 ± 0.04	0.46 ± 0.01	0.50 ± 0.02	0.76 ± 0.04	0.79 ± 0.03
IL-13 (pg/mL)	Air	0.26 ± 0.08	0.20 ± 0.09	0.10 ± 0.00	0.63 ± 0.08	0.66 ± 0.03
	PC	0.30 ± 0.22	0.17 ± 0.08	0.22 ± 0.12	0.54 ± 0.22	0.66 ± 0.07
IL-1β (pg/mL)	Air	2.59 ± 0.51	2.42 ± 0.26	2.04 ± 0.13	3.75 ± 0.46	3.63 ± 0.27
	PC	2.81 ± 0.61	2.28 ± 0.32	2.12 ± 0.19	3.71 ± 0.50	3.99 ± 0.28
IL-4 (pg/mL)	Air	0.34 ± 0.01	0.36 ± 0.03	0.35 ± 0.01	0.57 ± 0.02	0.55 ± 0.02
	PC	0.32 ± 0.03	0.34 ± 0.01	0.37 ± 0.02	0.58 ± 0.02	0.61 ± 0.05
IL-5 (pg/mL)	Air	8.60 ± 1.20	7.15 ± 1.37	4.28 ± 0.95	9.91 ± 2.69	7.79 ± 2.37
	PC	8.80 ± 1.37	6.32 ± 2.38	5.02 ± 2.50	11.42 ± 3.23	12.30 ± 1.97
IL-6 (pg/mL)	Air	142.42 ± 25.22	137.86 ± 18.42	132.64 ± 15.04	243.05 ± 38.51	181.60 ± 25.43
	PC	140.85 ± 11.41	147.62 ± 42.20	135.45 ± 22.94	267.54 ± 83.26	238.70 ± 25.91
KC (pg/mL)	Air	77.97 ± 9.66	88.06 ± 40.12	87.37 ± 30.28	177.38 ± 51.54	109.68 ± 15.23
	PC	68.89 ± 27.31	83.90 ± 17.18	71.62 ± 24.66	142.46 ± 27.17	131.03 ± 30.07
TNF-α (pg/mL)	Air	0.32 ± 0.28	0.44 ± 0.22	0.60 ± 0.08	0.67 ± 0.39	0.60 ± 0.15
	PC	0.39 ± 0.26	0.60 ± 0.36	0.55 ± 0.34	0.96 ± 0.54	0.90 ± 0.29

Values represent means ± SEMs; *n* = 8/group/time point.

* *p* < 0.05 versus air control group.

**TABLE 4.** Hematological Parameters

Parameter	Treatment	Exposure duration (days)				
		1	4	8	15	30
Erythrocytes (M/μL)	Air	9.75 ± 1.96	5.57 ± 0.35	6.70 ± 0.33	6.96 ± 0.34	7.24 ± 0.46
	PC	10.25 ± 2.06	6.32 ± 0.21	7.210. ± 17	7.07 ± 0.14	7.75 ± 0.32
Hemoglobin (g/dL)	Air	9.75 ± 1.96	11.26 ± 0.64	12.98 ± 0.58	13.12 ± 0.59	12.88 ± 0.70
	PC	10.25 ± 2.06	12.73 ± 0.36	13.82 ± 0.29	13.53 ± 0.22	13.50 ± 0.61
Hematocrit (%)	Air	28.13 ± 5.7	32.33 ± 2.12	37.80 ± 1.86	36.95 ± 1.87	36.83 ± 2.43
	PC	29.93 ± 5.99	37.55 ± 1.28	40.48 ± 1.03	38.85 ± 0.79	39.08 ± 2.07
MCV (fL)	Air	55.83 ± 1.41	58.03 ± 0.25	56.47 ± 0.76	53.08 ± 0.49	50.90 ± 0.58
	PC	56.51 ± 1.35	59.38 ± 0.49*	56.15 ± 0.42	54.95 ± 0.18*	50.30 ± 1.00
MCH (pg)	Air	19.26 ± 0.57	20.28 ± 0.24	19.42 ± 0.18	18.87 ± 0.19	17.87 ± 0.30
	PC	16.53 ± 3.31	20.15 ± 0.19	19.17 ± 0.13	19.17 ± 0.11	17.45 ± 0.18
MCHC (g/dL)	Air	34.45 ± 0.29	34.93 ± 0.42	34.40 ± 0.37	35.55 ± 0.34	35.13 ± 0.45
	PC	28.58 ± 5.71	33.96 ± 0.31	34.15 ± 0.17	34.85 ± 0.15	34.65 ± 0.38
RDW-CV (%)	Air	15.41 ± 0.44	15.38 ± 0.42	15.50 ± 0.46	16.28 ± 0.46	19.25 ± 0.68
	PC	15.51 ± 0.274	14.73 ± 0.16	15.82 ± 0.47	15.72 ± 0.29	19.37 ± 0.77
Reticulocytes (K/μL)	Air	6.29 ± 0.25	357.23 ± 29.07	279.30 ± 11.05	194.30 ± 15.40	210.98 ± 15.98
	PC	5.76 ± 0.34	354.82 ± 4.85	269.53 ± 8.72	199.62 ± 8.69	221.12 ± 13.49
Reticulocytes (%)	Air	6.29 ± 0. 75	6.43 ± 0.33	4.20 ± 0.19	2.78 ± 0.13	2.92 ± 0.16
	PC	5.76 ± 0.34	5.64 ± 0.22	3.74 ± 0.05	2.84 ± 0.16	2.85 ± 0.12
Platelets (K/μL)	Air	130.00 ± 112.41	624.17 ± 197.74	603.67 ± 139.26	656.17 ± 141.39	578.33 ± 148.26
	PC	574.8 ± 185.7	748.8 ± 162.71	469.67 ± 134.21	482.3 ± 112.04	538.5 ± 166.323
PDW (fL)	Air	7.83 ± 0.88	8.28 ± 0.25	8.08 ± 0.18	8.42 ± 0.19	8.66 ± 0.26
	PC	7.8 ± 0.19	7.96 ± 0.09	8.10 ± 0.22	8.33 ± 0.14	8.34 ± 0.30
MPV (fL)	Air	6.8 ± 0.61	6.98 ± 0.11	7.02 ± 0.12	7.14 ± 0.07	7.16 ± 0.16
	PC	6.7 ± 0.1	6.98 ± 0.06	7.18 ± 0.07	7.23 ± 0.14	6.96 ± 0.09
Leukocytes (K/μL)	Air	3.11 ± 0.78	4.05 ± 0.83	4.97 ± 0.53	4.74 ± 0.57	7.42 ± 1.06
	PC	3.83 ± 0.92	5.02 ± 0.58	5.34 ± 0.52	5.07 ± 0.61	6.32 ± 0.33
% Neutrophils	Air	7.13 ± 1.96	10.30 ± 0.75	12.68 ± 0.89	9.83 ± 0.85	14.23 ± 1.86
	PC	12.4 ± 2.94	9.78 ± 0.90	10.03 ± 0.89	10.33 ± 0.90	10.67 ± 0.53
% Lymphocytes	Air	89.22 ± 2.59	70.58 ± 13.82	82.15 ± 2.11	84.67 ± 0.99	79.45 ± 2.39
	PC	82.38 ± 3.26	85.82 ± 1.47	85.03 ± 1.32	82.98 ± 2.11	85.32 ± 0.71
% Monocytes	Air	2.68 ± 0.68	2.12 ± 0.21	3.62 ± 0.92	3.18 ± 0.37	2.13 ± 0.43
	PC	3.44 ± 0.53	3.22 ± 0.76	2.99 ± 0.69	2.15 ± 0.11*	2.13 ± 0.26
%Eosinophils	Air	0.87 ± 0.40	2.72 ± 1.43	1.33 ± 0.17	1.88 ± 0.52	3.98 ± 1.41
	PC	1.62 ± 0.33	1.1 ± 1.34	1.7 ± 0.28	4.23 ± 1.43	1.65 ± 0.22
% Basophils	Air	0.10 ± 0.07	0.12 ± 0.05	0.22 ± 0.07	0.43 ± 0.06	0.20 ± 0.10
	PC	0.16 ± 0.04	0.15 ± 0.05	0.25 ± 0.09	0.33 ± 0.10	0.23 ± 0.06

Values represent means ± SEMs; *n* = 8/group/time point.

* *p* < 0.05 versus air control group.

**TABLE 5.** Serum Chemistry Profiles

		Exposure duration (days)					
Marker	Treatment	1	4	8	15	30	Standard ranges
CREA (mg/dL)	Air	0.2 ± 0.02	0.1 ± 0.02	0.3 ± 0.02	0.2 ± 0.02	0.3 ± 0.02	0.1–0.7
	PC	0.1 ± 0.02*	0.2 ± 0.02	0.3 ± 0.02	0.2 ± 0.02	0.25 ± 0.02	
BUN (mg/dL)	Air	13 ± 0.45	13 ± 0.71	15 ± 0.89	14 ± 0.49	16 ± 0.60	9–21
	PC	12 ± 0.73	13 ± 0.86	14 ± 0.58	15 ± 0.87	16 ± 1.0	
PHOS (mg/dL)	Air	9.7 ± 0.37	8.7 ± 0.30	10.0 ± 0.386	8.2 ± 0.18	7.6 ± 0.27	5.8–11.2
	PC	10.05 ± 0.18	9.87 ± 0.50	10.3 ± 0.488	7.9 ± 0.31	7.4 ± 0.20	
TP (g/dL)	Air	5.1 ± 0.11	5.2 ± 0.21	5.5 ± 0.13	5.60.11 ±	9.0 ± 0.08	5.3–6.9
	PC	5.1 ± 0.08	5.1 ± 0.1	5.5 ± 0.082	5.6 ± 0.052	9.3 ± 0.11	
ALB (g/dL)	Air	2.6 ± 0.04	3.1 ± 0.10	2.8 ± 0.061	2.7 ± 0.052	2.8 ± 0.048	3.8–4.8
	PC	2.7 ± 0.06	3.0 ± 0.16	2.8 ± 0.093	2.7 ± 0.048	2.9 ± 0.10	
GLOB (g/dL)	Air	2.5 ± 0.08	4.8 ± 2.7	2.7 ± 0.070	2.9 ± 0.077	3.1 ± 0.048	15–2.8
	PC	2.5 ± 0.03	2.1 ± 0.24	2.7 ± 0.037	2.9 ± 0.040	3.1 ± 0.060	
ALT (U/L)	Air	40 ± 3.7	39 ± 6.4	51 ± 3.9	34 ± 1.6	37 ± 2.9	20–61
	PC	38.8 ± 3.5	39 ± 5.5	51 ± 3.9	38 ± 2.5	44 ± 7.9	
ALKP (U/L)	Air	231 ± 11.7	252 ± 14.4	207 ± 8.38	220 ± 19.1	155 ± 10.2	16–302
	PC	218 ± 13.5	261 ± 24.8	218 ± 9.04	188 ± 9.55	161 ± 9.40	
TBIL (mg/dL)	Air	0.1 ± 0.02	0.2 ± 0.06	0.1 ± 0.03	0.1 ± 0.03	0.2 ± 0.07	0.1–0.7
	PC	0.12 ± 0.02	0.15 ± 0.03	0.2 ± 0.03	0.1 ± 0.0	0.2 ± 0.1	
CHOL (mg/dL)	Air	42 ± 4.5	34 ± 5.7	38 ± 2.3	38 ± 3.3	36 ± 2.2	20–92
	PC	42 ± 1.5	41 ± 4.3	35 ± 2.7	40 ± 3.1	32 ± 5.8	
CK (U/L)	Air	726 ± 214	1067 ± 108.0	1038 ± 235.8	1021 ± 93.68	906 ± 102.9	48–340
	PC	891 ± 241	921.2 ± 155.8	1061 ± 75.18	1026 ± 95.40	1010 ± 210.1	
LDH (U/L)	Air	2751 ± 636.6	6226 ± 625.8	4113 ± 491.1	6990 ± 578.3	4557 ± 672.8	167–1428
	PC	4592 ± 1202	4163 ± 1006	5561 ± 313.8*	5878 ± 544.1	4504 ± 882.1	
URIC (mg/dL)	Air	0.53 ± 0.08	0.7 ± 0.04	1.3 ± 0.64	0.5 ± 0.09	0.5 ± 0.06	0.8–4.4
	PC	0.80 ± 0.23	1.1 ± 0.44	1.3 ± 0.71	0.4 ± 0.05	0.4 ± 0.06	
AST (U/L)	Air	79.8 ± 10.3	91.24.45 ±	91.8 ± 11.8	92 ± 3.3	94 ± 7.0	39–111
	PC	119 ± 39.1	77 ± 6.0	99.8 ± 6.62	90 ± 3.6	101 ± 12.3	
NH3 (μmol/L)	Air	20 ± 3.72	32 ± 4.1	59 ± 23	36 ± 4.7	34 ± 4.2	N/A
	PC	38 ± 7.7	40 ± 13	70 ± 27	28 ± 3.2	33 ± 7.2	
CRP (mg/dL)	Air	0.25 ± 0.02	0.2 ± 0.0	0.2 ± 0.03	0.3 ± 0.02	0.3 ± 0.03	N/A
	PC	0.30 ± 0.09	0.22 ± 0.02	0.2 ± 0.0	0.3 ± 0	0.3 ± 0.02	

Values represent means ± SEMs; *n* = 8/group/time point.

* *p* < 0.05 versus air control group.
